# Adrenergic Signaling at the Interface of Allergic Asthma and Viral Infections

**DOI:** 10.3389/fimmu.2018.00736

**Published:** 2018-04-11

**Authors:** Didem Ağaç, Michelle A. Gill, J. David Farrar

**Affiliations:** ^1^Department of Immunology, The University of Texas Southwestern Medical Center, Dallas, TX, United States; ^2^Department of Pediatrics, The University of Texas Southwestern Medical Center, Dallas, TX, United States

**Keywords:** adrenergic receptor, asthma, rhinovirus, inflammation, cytokine

## Abstract

Upper respiratory viral infections are a major etiologic instigator of allergic asthma, and they drive severe exacerbations of allergic inflammation in the lower airways of asthma sufferers. Rhinovirus (RV), in particular, is the main viral instigator of these pathologies. Asthma exacerbations due to RV infections are the most frequent reasons for hospitalization and account for the majority of morbidity and mortality in asthma patients. In both critical care and disease control, long- and short-acting β2-agonists are the first line of therapeutic intervention, which are used to restore airway function by promoting smooth muscle cell relaxation in bronchioles. While prophylactic use of β2-agonists reduces the frequency and pathology of exacerbations, their role in modulating the inflammatory response is only now being appreciated. Adrenergic signaling is a component of the sympathetic nervous system, and the natural ligands, epinephrine and norepinephrine (NE), regulate a multitude of autonomic functions including regulation of both the innate and adaptive immune response. NE is the primary neurotransmitter released by post-ganglionic sympathetic neurons that innervate most all peripheral tissues including lung and secondary lymphoid organs. Thus, the adrenergic signaling pathways are in direct contact with both the central and peripheral immune compartments. We present a perspective on how the adrenergic signaling pathway controls immune function and how β2-agonists may influence inflammation in the context of virus-induced asthma exacerbations.

## Rhinovirus (RV)-Induced Asthma Exacerbations

Asthma is a debilitating chronic disease that has a significant impact on society, including decreased quality of life, work productivity, and increased utilization of health-care resources. With total annual costs reported at $81.9 billion in the U.S. alone (1), asthma represents an enormous economic burden. Approximately 2 million annual emergency room visits and 500,000 hospitalizations have been attributed to acute asthma management in the U.S. (2), highlighting the substantial contribution of asthma exacerbations to the morbidity associated with this disease. Respiratory viral infections are commonly associated with asthma exacerbation episodes (3–6), and RVs have long been recognized as the most frequent viral contributors. The seasonality of RV-associated asthma exacerbations has also been well described, with predictable peaks of hospitalizations for asthma occurring during September epidemics of RV infection (7).

The mechanisms underlying this association between RV and exacerbations of asthma represent an area of intense investigation. The impact of the infection itself on the lung represents one potential mechanism. Although most commonly detected in upper airway samples, RVs have also been demonstrated in lower airway fluids and cells following experimental infection of the upper airway (8–10). Paired with clinical evidence linking RV to lower respiratory tract infections in children (11–13), it is possible that RV infection directly injures airway tissues in the lower airway (14), potentially contributing to exacerbations of asthma. RV infection of airway epithelial cells (ECs) induces the expression of a range of chemokines and cytokines that promote ensuing inflammatory responses. These include such pro-inflammatory molecules as IL-8/CXCL8 (15–17), IL-6 (17, 18), CCL11/eotaxin-1, RANTES/CCL5 (19), IP-10/CXCL10 (20), and ICAM-1 (21). In turn, inflammatory cells recruited by these chemokines secrete IFN-γ and TNF-α, which in some cases can provide a direct antiviral activity in target cells mimicking type I interferon (22). Increased concentrations of inflammatory cytokines have also been demonstrated in airway samples (nasal samples and sputum) obtained from RV-infected individuals (21, 23, 24). RV-induced secretion of such chemokines may also promote asthma exacerbations by promoting an influx of immune cells such as eosinophils, neutrophils, lymphocytes, and macrophages (Mɸs) into the airway (25). Immune cells themselves have also been shown to contribute to the epithelial RV response; human monocytic cells amplify bronchial epithelial cell (BEC) chemokine production during RV infection (26) and could thus also influence asthma pathogenesis in the setting of RV infection.

In addition to the chemokines listed above, RV also induces type I IFN (IFN) expression in airway ECs. The demonstration of decreased IFN-β responses in RV-infected BECs from asthmatics led to the hypothesis that defective IFN antiviral responses could contribute to the pathogenesis of asthma exacerbations (27). While virtually all somatic cells have the capacity to produce IFN-α/β in response to infection, specialized plasmacytoid dendritic cells (pDCs) are the primary cell type to secrete IFN at high levels in response to viral infection. Furthermore, human pDCs also express the high affinity IgE receptor, enabling them to respond to both viral and allergic signals. Deficient viral-induced IFN responses have been demonstrated in virus-simulated whole-blood cultures (28, 29) and purified pDCs (30) from individuals with allergic asthma, providing further evidence for a potential role of IFN in asthma exacerbations. In addition, the link between IgE and pDC antiviral IFN responses could explain the increased risk of asthma exacerbations seen in the presence of atopy and respiratory viral infections. Allergic sensitization and elevated IgE levels are known risk factors for asthma exacerbations with RV infection (3). The magnitude of pDC IFN responses to *in vitro* viral challenge is inversely correlated with serum IgE levels. In addition, IgE cross-linking abrogates viral-induced pDC IFN production (30, 31). In a recent NIAID-sponsored trial of omalizumab in children with allergic asthma, RV-induced pDC IFN responses were significantly increased in the group who received this IgE-reducing treatment, and this improved antiviral response was associated with lower exacerbations (31, 32).

Since pDCs represent the major source of this antiviral cytokine (33), a defect in IFN production, this cell type could explain how viral infection promotes severe disease in patients with asthma. Another potentially significant effect of reduced pDC antiviral IFN production includes the effect on T helper type 2 responses. IFN has recently been shown to reverse the Th2 phenotype of CD4 lymphocytes *via* suppression of the Th2 transcription factor GATA-3 (34, 35) and to acutely inhibit IL-5 and IL-13 secretion from memory Th2 cells (36). Thus, a deficient IFN response during respiratory RV infection could contribute to the increased Th2 inflammation observed in individuals with allergic asthma.

## Control of Immune Function by Adrenergic Signaling

While the use of corticosteroids and long-term β2-agonists are used for maintenance therapy for asthma sufferers, the front-line intervention for acute exacerbations driven by RV infections is the short-acting β2-agonist, ventolin (nebulized albuterol). The β2-adrenergic receptor (ADRB2) is expressed on smooth muscle cells surrounding the bronchioles, and activation of this receptor by both the natural ligand, epinephrine and norepinephrine (NE), as well as β2-agonists promotes smooth muscle cell relaxation and restored breathing capacity. Signaling through adrenergic receptors controls a myriad of physiological responses, including heart rate, respiratory capacity, and lung turgor. As such, both natural and synthetic ligands for adrenergic receptors have been chiefly used to control sepsis, heart disease, COPD, and asthma.

Innervating throughout most tissues and organs, post-ganglionic sympathetic neurons release the major neurotransmitter NE in response to various intrinsic and external stimuli. Diurnal fluctuations in the release of NE link the sympathetic nervous system to circadian rhythms. Sympathetic neurons also control the “fight or flight” response during periods of stress or fear. Upon ligand binding, adrenergic receptors can activate various G-proteins, depending upon the class of receptor and the specific cell types that express them. For example, the binding of adrenaline and noradrenaline to β2AR results in activation of Gαs (the stimulatory subunit of heterotrimeric G protein) and subsequently activation of adenylyl cyclase, increase in cyclic AMP (cAMP) concentration, and activation of cAMP-dependent protein kinase A (PKA). Depending on the cell that the receptor is engaged, PKA activation can lead to several physiological changes, including muscle contraction, cytokine secretion, and so on. Moreover, the same receptor can couple to the inhibitory Gαi and or signal through MAP kinase pathways (37–39). This complex behavior of the adrenergic receptors enables these receptors to induce cell- and context-specific physiological changes.

**Figure 1:**
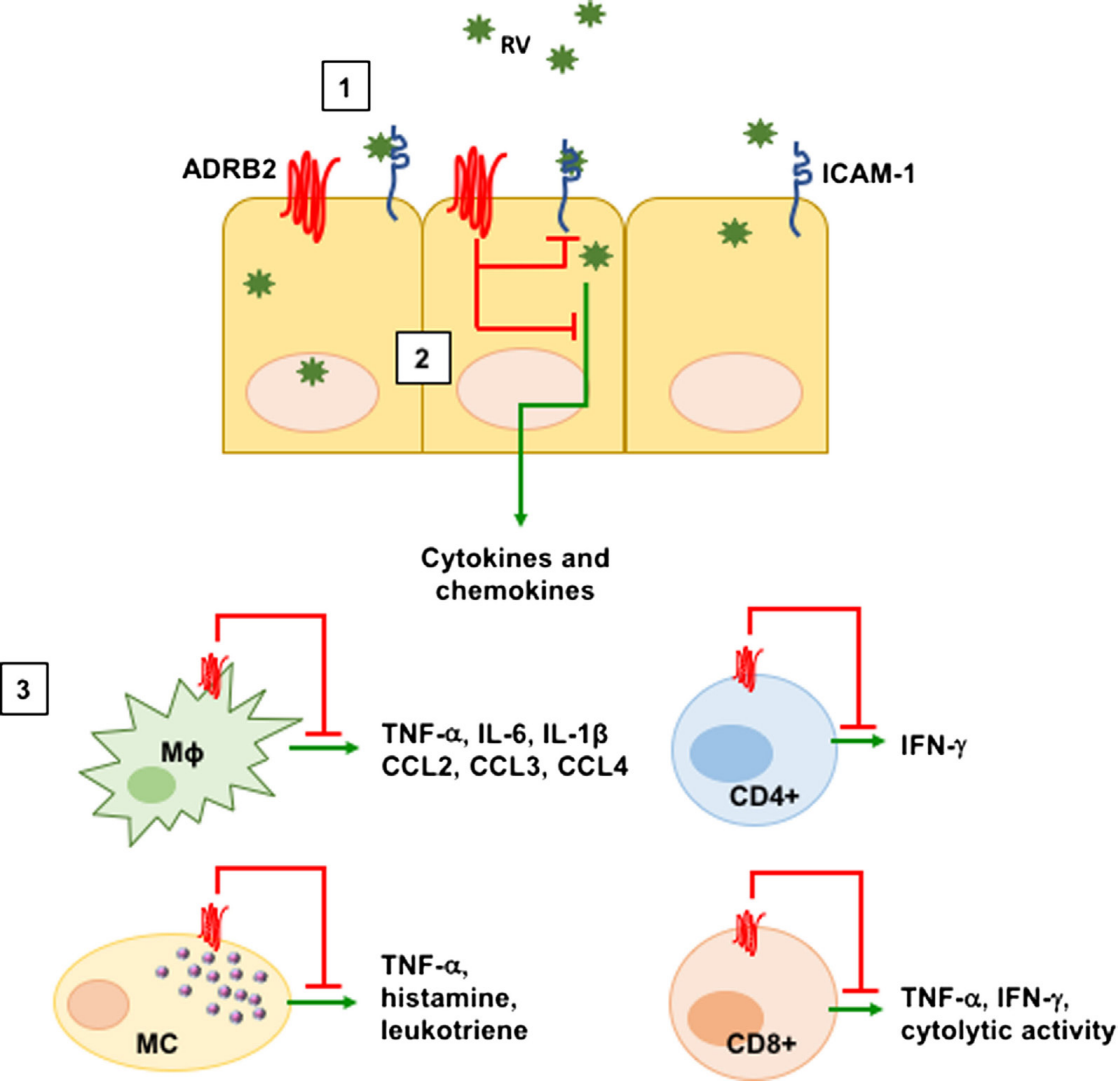
ADRB2-mediated suppression of inflammatory processes. Adrenergic signaling through the ADRB2 inhibits various virus-induced immune mediators. [1] Rhinovirus (RV) infects the upper airways by binding to ICAM-1 on the surface of lung ECs. RV infection of ECs upregulates ICAM-1 as well as IL-8, IL-6, CCL5, CCL11, and CXCL10 to recruit inflammatory cells. [2] Activation of the ADRB2 by either the natural ligands epinephrine and norepinephrine or by β2-agonists downregulates ICAM-1 as well as IL-8, CCL5, and GM-CSF from ECs. [3] ADRB2 signaling additionally inhibits pro-inflammatory mediators in innate and adaptive immune cells. Abbreviations: EC, epithelial cell; MC, mast cell; Mɸ, macrophage.

The ADRB2 is expressed widely on many types of immune cells, albeit at different levels of cell surface ligand binding sites (40). For example, Maisel et al. identified expression of beta-adrenergic receptor density on lymphocytes ranging from 1,000 to 2,000 receptors/cell (41). In general, ADRB2 signaling acts to suppress the level of inflammation and cytokine secretion in both innate and adaptive T cells (diagrammed in Figure 1). For example, recent studies demonstrated that CD8^+^ T cell effector function was impaired in response to adrenergic receptor signaling (42–44). Presence of β2-agonists such as albuterol reduced TCR-induced IFNγ and TNFα production, as well as cytolytic activity of both human and murine T cells (43). Similarly, use of beta-blockers increased the frequency of intratumoral CD8^+^ T cells and increased the efficacy of anti-PD-1 treatment (45). In CD4^+^ T cells, the presence of NE increases IFN-γ production from Th1 cells (46). Although Th1 cells have been reported to be affected by NE, Th2 cells are less responsive to NE due to the reduction of ADRB2 expression during differentiation and lack of the receptor expression on mature Th2 cells (47, 48). In addition to suppressing T cell effector function, previous studies have demonstrated that ADRB2 signaling can also inhibit TNF-α and IL-12 secretion from innate cells including dendritic cells and Mɸs (49–52) perhaps through direct inhibition of TLR-mediated NF-κB activation (53, 54). Finally, ADRB2 signaling has been shown to enhance the suppressive function of Treg cells (55), which may have significance for clinical effectiveness in asthma.

## Role of β2-Agonists in the Context of RV-Mediated Inflammation

Rhinovirus infects human airway ECs by binding to ICAM-1 (Figure 1). As discussed earlier, the natural course of inflammation and cytokine expression increases ICAM-1 expression, allowing additional migration of inflammatory cells into sites of infection. This process likely contributes to RV-induced exacerbations in allergic subjects. Interestingly, both natural ligands of adrenergic receptors (epinephrine and NE), as well as synthetic agonists of the ADRB2 (salbutamol and terbutaline) downregulate ICAM-1 expression on monocytes (56, 57). Furthermore, human BECs reduced the expression of ICAM-1 in response to fenoterol, a β2-agonist (58), suggesting the use of β2-agonists might help patients with RV-induced exacerbations by downregulating the entry receptor on various cell types. Moreover, human airway parasympathetic neurons also downregulate expression of ICAM-1 (59), raising the possibility that the use of β2-agonists has a broader effect than previously appreciated.

Smooth muscle cell responsiveness to β2-agonists is critical for emergency intervention during exacerbations. However, RV infection has been shown to reduce expression of the ADRB2 on airway smooth muscle cells *via* indirect actions on infected ECs. RV drives secretion of prostaglandins from ECs, which act in a paracrine fashion on smooth muscle cells to suppress ADRB2 expression (60). In this context, COX2 inhibitors tended to restore adrenergic responsiveness. Airway ECs express functional adrenergic receptors (61, 62). Stimulation of the ADRB2 increased the beat frequency of cilia (63), and fenoterol, a β2-agonist downregulates ICAM-1 (58). Sabatini and colleagues reported that salmeterol downregulated VCAM-1 in addition to ICAM-1. In addition, RANTES, IL-8, and GM-CSF were inhibited in response to adrenergic stimulation (64). In murine models, airway EC-specific expression of ADRB2 can recapitulate IL-13-induced airway hyperresponsiveness, mucus production, and cellular infiltration (65), which contrasts to the suppressive effects of β2-agonists seen in human cells.

Victoni et al. demonstrated that β2-agonists can downregulate TNFα, IL-6, and IL-1β from human monocyte-derived Mɸs; however, lung Mɸs are resistant to suppressive effects of β2-agonists (66). Similarly, chemokines CCL2, CCL3, and CCL4 were downregulated in human monocyte-derived Mɸs, yet lung Mɸs were not affected. One possible mechanism of cytokine suppression may involve targeting cytokine mRNA transcripts. For example, β2-agonist salbutamol increases the expression of tristetraproline (TTP) in murine and human Mɸ cell lines. TTP can bind to AU-rich elements in 3′UTR of several pro-inflammatory cytokine transcripts, including TNF and GM-CSF. This interaction might account for the reduction in pro-inflammatory cytokines in response to adrenergic signaling (67). Although lung Mɸs had similar levels of ADRB2 transcript, the ADRB2 protein was not expressed, which can explain why lung Mɸs may not respond to β2-agonists as efficiently as their splenic and circulating counterparts (66). β2-Agonists inhibit release of histamine and leukotriene from mast cells (MCs) *in vitro* and *in vivo* (68–70). Similarly, β2-agonists reduce histamine release from human lung MCs when cocultured with airway smooth muscle cells (71). IgE-mediated release of TNFα is also reduced in response to β2-agonists (72). These findings suggest that MC mediators that are involved in acute inflammatory responses can be controlled by adrenergic receptor agonists.

In IFN-γ-primed human dendritic cells, salbutamol inhibited IL-12, IL-1α, IL-1β, IL-6, and TNFα; however, IL-10 was unaffected. When naive T cells were primed with dendritic cells exposed to salbutamol, commitment to Th1 lineage significantly reduced (possibly due to the reduction in IL-12) (49). This is accompanied by an increase in IL-4^+^ Th2 cells in the coculture. This suggests that use of β2-agonists may skew lung T cells to the pathogenic Th2 lineage. Similarly, in murine bone marrow-derived dendritic cells, epinephrine enhanced differentiation of IL-4- and IL-17A-producing T cells (73). In addition to T cell priming, β2-agonists also alter phagosomal degradation of antigens and cross-presentation of dendritic cells (74). Finally, Yewdell and colleagues recently demonstrated that chemical sympathectomy increased CD8^+^ T cell responses to influenza infection in mice (42). Furthermore, ADRB2 antagonists enhanced CD8^+^ responses, and while a direct role for the ADRB2 on CD8^+^ T cells was not examined, this study suggests that adrenergic signaling acts to limit the response to viral infections.

## Final Comments and Future Areas of Interest

Although β2-agonists are widely used in the management of asthma and COPD, many questions remain regarding their ability to suppress inflammation in the context of exacerbations. As mentioned earlier, stimulation of ECs *in vitro* with β2-agonists downregulates ICAM-1 expression. This indicates that the use of β2-agonists can potentially reduce RV entry and spread within the lungs. Yamaya and colleagues reported that pretreatment of human tracheal ECs with tulabuterol, a long-acting β2-agonist, for 3 days before RV-14 exposure reduced the expression of ICAM-1 and viral replication in ECs (75). By contrast, Bochkov and colleagues reported that pretreatment of BECs with budesonide (a corticosteroid), formoterol (a β2-agonist), or in combination for 24 h did not alter replication of RV-16 in asthmatics and healthy subjects (76). However, the authors did not present data on the level of ICAM-1 protein. It would be beneficial to assess the role of β2-agonists *ex vivo* during RV infections to eliminate the variation from *in vitro* settings. Also, no studies to date have investigated the role of adrenergic receptor signaling on expression of RV viral proteins. Moreover, β2-agonists promote an anti-inflammatory phenotype in innate and adaptive immune cells by suppressing production of antiviral cytokines (43) and downregulate a plethora of chemokines (64) that can contribute to recruitment of inflammatory cells to the lungs. This raises the issue of the benefits versus costs of the use of long-term β2-agonists to control asthma symptoms. If β2-agonists generally suppress innate immune function, does their use allow for a more receptive environment for infection? By contrast, in the contest of overt RV-driven inflammation, β2-agonists can certainly dampen the magnitude of inflammation, which is also thought to be the main benefit of corticosteroids. Additional studies are warranted to determine the long-range effects of β2-agonists in the context of both RV susceptibility and the acute effects these drugs have on suppressing inflammation during exacerbations.

## Author Contributions

DA wrote drafts of Sections “Control of Immune Function by Adrenergic Signaling” and “Role of β2-Agonists in the Context of RV-Mediated Inflammation.” MG wrote Section “Rhinovirus (RV)-Induced Asthma Exacerbations.” JF conceived of the subject, wrote the abstract, and edited the manuscript.

## Conflict of Interest Statement

The authors declare that the research was conducted in the absence of any commercial or financial relationships that could be construed as a potential conflict of interest.
